# Supplementary material for: Inhibition Studies on Human and Mycobacterial Carbonic Anhydrases with N-((4-Sulfamoylphenyl)carbamothioyl) Amides
Source: Molecules. 2023 May 11;28(10):4020. doi: 10.3390/molecules28104020 (PMC10222120; doi:10.3390/molecules28104020)
Supplement: Supplementary file 1 [file molecules-28-04020-s001.zip › molecules-2264740-supplementary.pdf]

Supporting Information

Inhibition studies on human and mycobacterial carbonic anhy-drases with N-((4-sulfamoylphenyl)carbamothioyl) amides

Morteza Abdoli, Alessandro Bonardi, Niccolò Paoletti, Ashok Aspatwar, Seppo Parkkila, Paola Gratteri, Claudiu T. Supuran and Raivis Žalubovskis

Sequence alignment of MtCA3 S2

3D representation of MtCA3 homology model S3

Structural parameters of MtCA3 homology model and related template S4

2D representation of benzenesulfonamide binding mode in MtCAs active sites S5

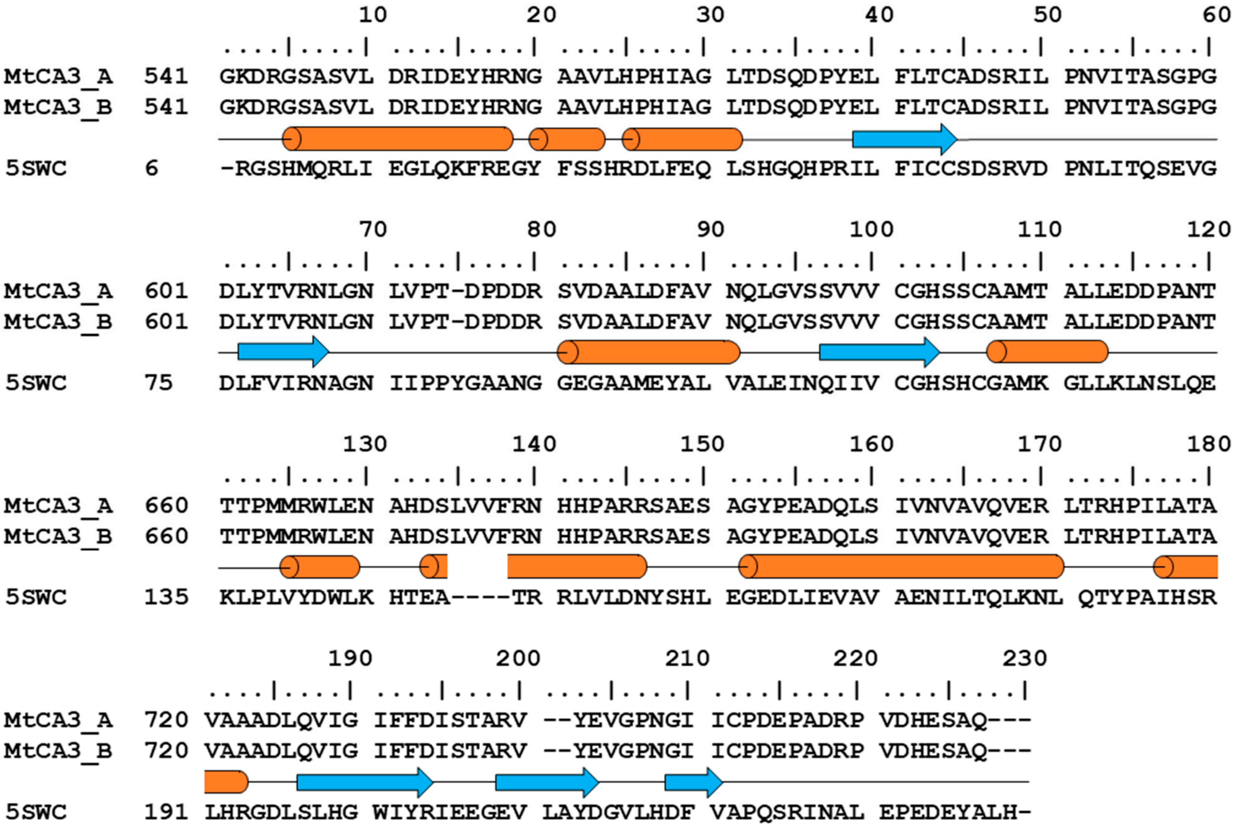

Figure S1. Sequence alignment of MtCA3 with the template β-CA from *Synechocystis* sp. PCC 6803 (pdb 5SWC).

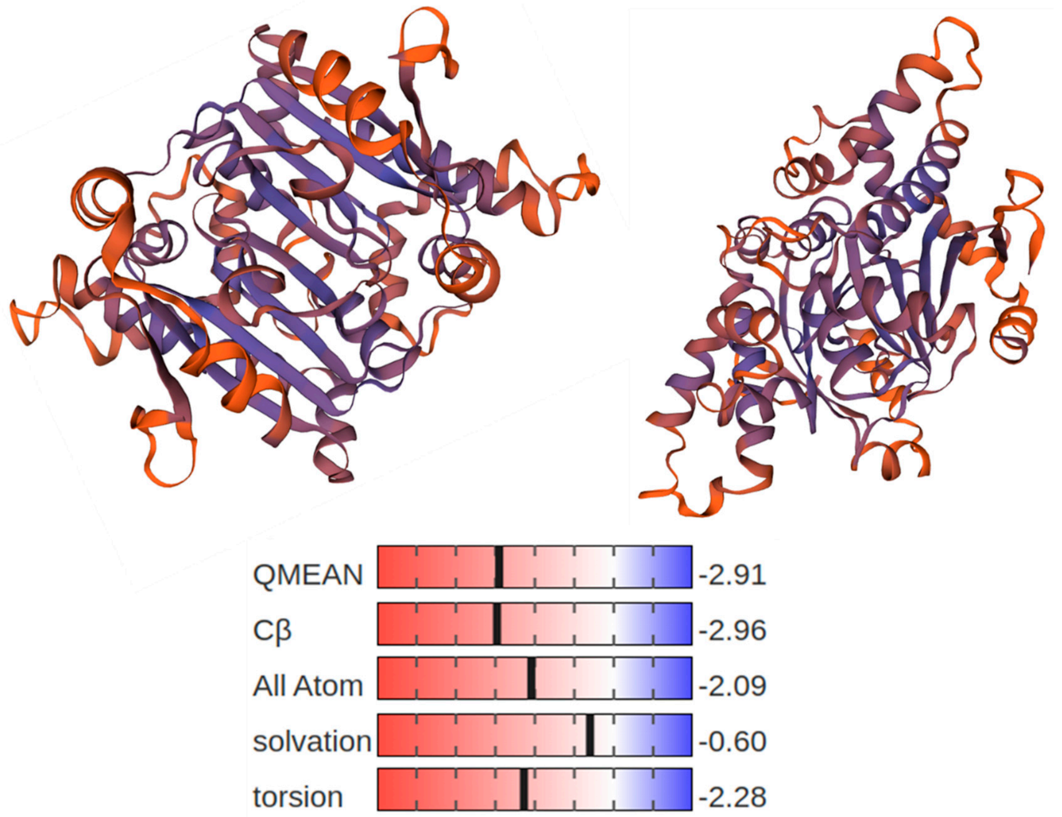

Figure S2. 3D representation of the homology model of type I MtCA3 and related parameters calculated from Swiss-model.

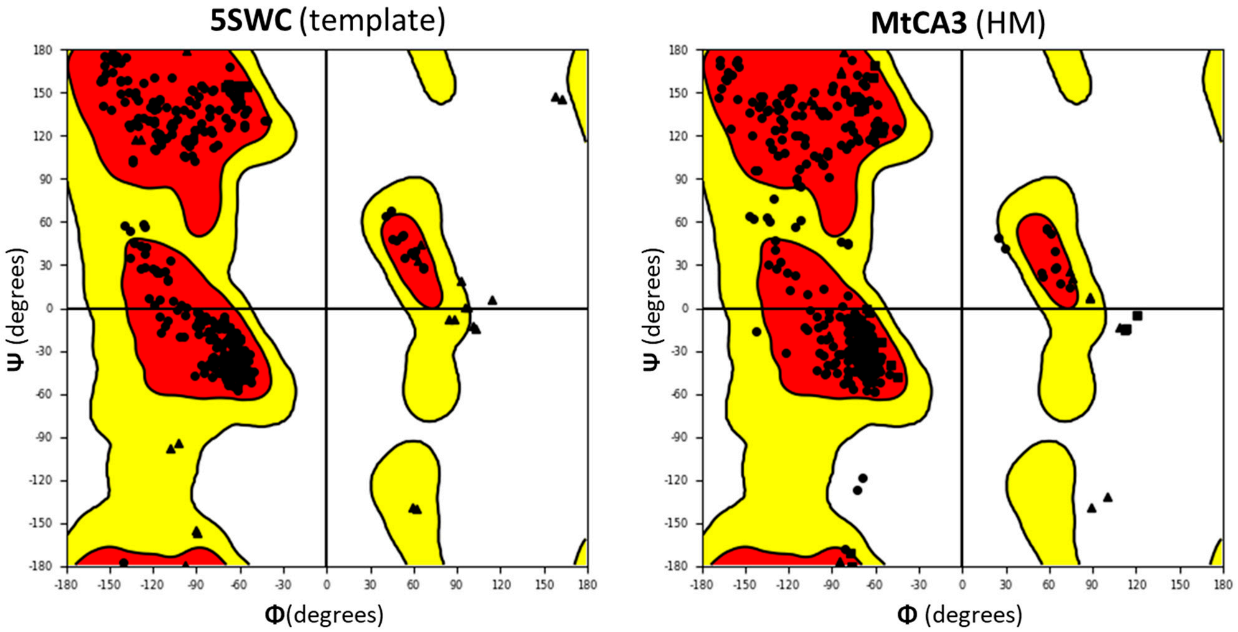

Figure S3. Ramachandran Plot of 5SWC (template) and MtCA3 (HM).

	5SWC	MtCA3
**MolProbity score**	1.22	1.71
**Clash score**	4.48	2.32
**Ramachandran Favoured**	99.38 %	94.53 %
**Ramachandran Outliers**	0.00 %	1.49 %
**Rotamer Outliers**	0.88%	2.68 %
**C-Beta Deviations**	0	8
**Bad Bonds**	0/3602	0/3138
**Bad Angles**	1/4886	59/4302
**QMEAN**	1.31	-2.91
**Cβ**	1.27	-2.96
**All Atom**	0.38	-2.09
**solvation**	0.98	-0.60
**torsion**	0.76	-2.28

Table S1. Structural parameters of 5SWC (template) and MtCA3 (HM).

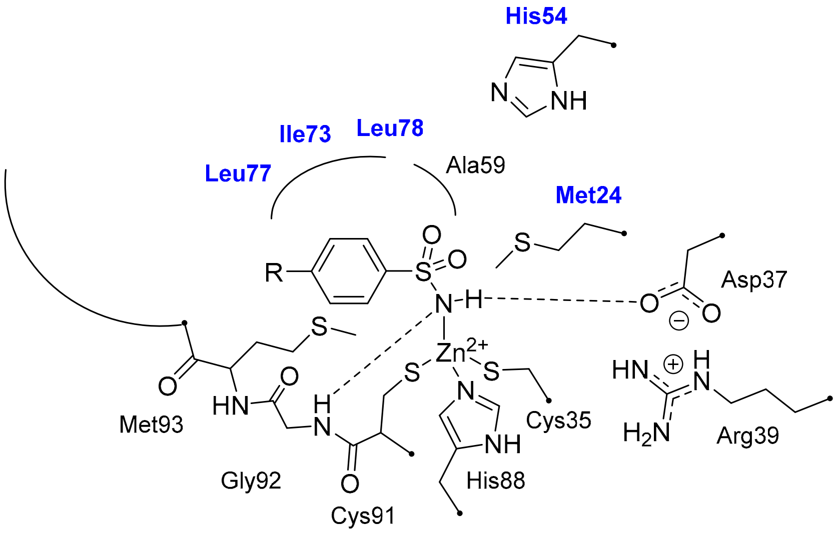

Figure S4. 2D schematic representation of MtCA1 residues involved in the interaction with benzenesulfonamides hypothesized by the *in silico* studies. The labels of amino acids from different chains are colored differently.

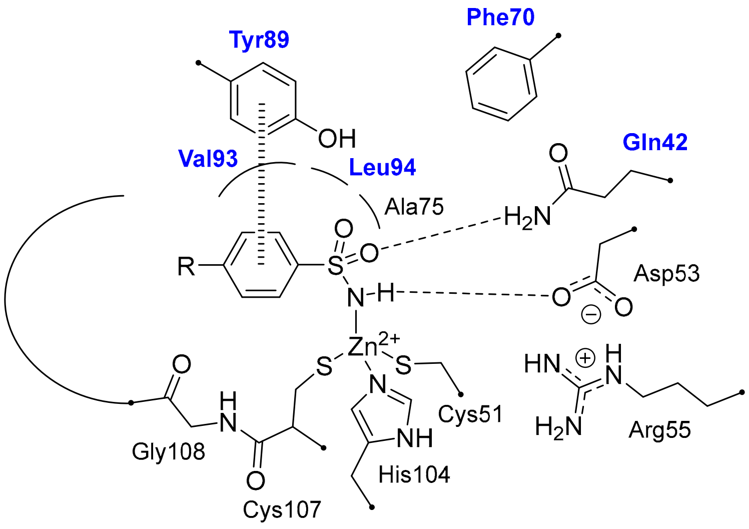

Figure S5. 2D schematic representation of MtCA2 residues involved in the interaction with benzenesulfonamides hypothesized by the *in silico* studies. The labels of amino acids from different chains are colored differently.

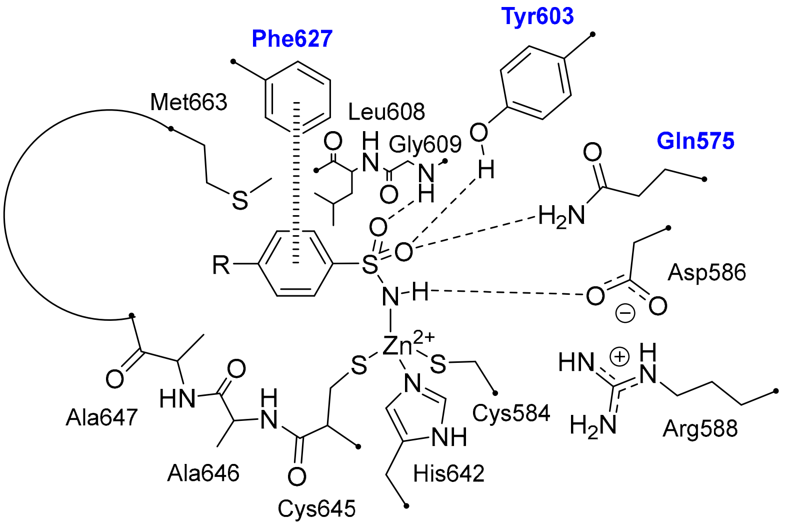

Figure S6. 2D schematic representation of MtCA3-HM residues involved in the interaction with benzenesulfonamides hypothesized by the *in silico* studies. The labels of amino acids from different chains are colored differently.
